# A Novel Major Facilitator Superfamily Transporter Gene *Aokap4* near the Kojic Acid Gene Cluster Is Involved in Growth and Kojic Acid Production in *Aspergillus* *oryzae*

**DOI:** 10.3390/jof8080885

**Published:** 2022-08-22

**Authors:** Tianming Chen, Ziming Chen, Yuzhen Li, Bin Zeng, Zhe Zhang

**Affiliations:** 1 Jiangxi Key Laboratory of Bioprocess Engineering and Co-Innovation Center for In-Vitro Diagnostic Reagents and Devices of Jiangxi Province, College of Life Sciences, Jiangxi Science and Technology Normal University, Nanchang 330013, China; 2 College of Pharmacy, Shenzhen Technology University, Shenzhen 518118, China

**Keywords:** *Aspergillus oryzae*, kojic acid, MSF transporter, kojic acid gene cluster

## Abstract

Kojic acid is an important secondary metabolite of industrial importance produced by *Aspergillus oryzae*. The kojic acid gene cluster plays an essential role in kojic acid production, and harbors *kojA*, *kojR* and *kojT*. The deletion of *kojT*, encoding a major facilitator superfamily (MFS) transporter, did not completely abolish kojic acid production, implying that other transporters are required for the transport of kojic acid. The aim of this study is to look for the transporters involved in kojic acid production. Here, *Aokap4* encoding a novel MFS transporter was identified, which was adjacent to *kojT* in the kojic acid gene cluster. The deletion of *Aokap4* contributed to the hyphal growth, conidial production and biomass of *A. oryzae*. Moreover, *Aokap4* is required for heat- and cell-wall-stress tolerance but not oxidative and osmotic stress. The disruption of *Aokap4* impaired kojic acid production with the reduced expression of *kojA*, *kojR* and *kojT*. Furthermore, when *kojT* was deleted in the *Aokap4*-disrupted strain, the yield of kojic acid declined to the same level as that of the *kojT*-deletion mutant, whereas the production of kojic acid was recovered when *kojT* was overexpressed in the *Aokap4* knockout strain, suggesting that *kojT* acts downstream of *Aokap4*. AoKap4 was the second identified MSF transporter involved in kojic acid production after *kojT* was found a decade ago. This study contributes to a better understanding of the biological roles of the MFS transporter and lays a foundation for future studies on kojic acid synthesis in *A. oryzae*.

## 1. Introduction

Kojic acid has great application value in such fields as the food, cosmetic, pharmaceutical and agriculture industries, and is a secondary metabolite produced by *Aspergillus oryzae* [1,2,3]. Some progress has been made in studying the mechanism underlying kojic acid synthesis [4,5,6,7,8,9,10]. The global transcriptional regulator of the secondary metabolite laeA negatively mediates kojic acid production through the regulation of the kojic acid synthesis genes *kojA*, *kojR* and *kojT* [4]. As the upstream of *laeA*, loss of function in the histone deacetylase gene *AoHst4* contributes to kojic acid biosynthesis, coupled with high levels of expression of *kojA*, *kojR* and *kojT* [6]. Additionally, some membrane proteins have been reported to be involved in kojic acid production, such as NrtA, AoZip2, AoKap1 and AoKap2 [5,11,12,13]. In *A. oryzae*, *nrtA* encodes a nitrate transporter, which abolishes the suppressive effect of nitrate on kojic acid production [5]. RNA interference of the ZIP transporter gene *AoZip2* inhibits kojic acid biosynthesis [12]. *Aokap1* and *Aokap2* both encode an uncharacterized protein with transmembrane domains, and the knockout of *Aokap1* or the overexpression of *Aokap2* contributes to kojic acid production [11,13]. These proteins affect kojic acid production via altered expression of *kojA*, *kojR* and *kojT* [4,11,12,13]. The kojic acid gene cluster of *A. oryzae* was identified a decade ago, and consists of three genes: *kojA*, *kojR* and *kojT*. However, little is known about the function of genes near the kojic acid gene cluster except for the reported kojic acid synthesis genes [7]. *KojR* is located in the middle of *kojA* and *kojT* [7]. The Zn(II)_2_Cys_6_ transcription factor KojR positively regulates kojic acid synthesis through the kojic acid synthesis enzyme KojA and the major facilitator superfamily (MFS) transporter KojT, and *kojR* is induced by kojic acid [8]. The disruption of *kojA* causes the complete loss of kojic acid production, whereas the deletion of *kojT* only results in the reduced secretion of kojic acid, suggesting that other transporters are required for transporting synthesized kojic acid to the extracellular environment [7]. However, little information on other MSF transporters involved in kojic acid, except for the KojT transporter in *A. oryzae*, is available to date.

The MFS transporters are one of the largest superfamilies of membrane transporters [14,15,16]. They have a broad spectrum of substrates, including sugars, polyols, metabolites and even inorganic tetraoxyanions [14,15,17]. Most MFS transporters harbor either 12 or 14 putative transmembrane domains (TM) [18]. According to the Transporter Classification Database (https://tcdb.org/ (accessed on 20 August 2022)), the MFS superfamily is divided into 61 families, including the sugar porter family. Many MFS transporters of the sugar porter family have been identified and characterized [18,19,20], for example, high-affinity glucose transporter HXT2, galactose transporter GAL2, glycerol transporter STL1, etc. [21,22,23,24,25]. However, very limited knowledge on MFS transporters is available for *A. oryzae*.

In this present study, the novel MFS transporter gene *Aokap4* (the kojic acid-related proteins *Aokap1*, *Aokap2* and *Aokap3* have already been studied, and *Aokap4* (AO090113000139) is going to be studied in this work) was identified near the kojic acid gene cluster, which was adjacent to *kojT*. The *Aokap4-*deletion strain was constructed using the CRISPR/Cas9 system. The phenotypes of the wild-type and *Aokap4*-disrupted strains were compared and analyzed. The effects of the deletion of *Aokap4* on kojic acid production were investigated. Moreover, double-deletion mutants of *Aokap4* and *kojT* were constructed to analyze their influence on kojic acid production. Meanwhile, the strain where *Aokap4* was deleted and *kojT* was overexpressed was generated to explore the relationship between *Aokap4* and *kojT*. Our data reveal that the *Aokap4* gene near the kojic acid gene cluster is required for growth, and for the responses to heat and cell-wall stress, and is involved in kojic acid production acting upstream of *kojT*. AoKap4 was the second identified MSF transporter required for kojic acid production after the finding of *kojT* within the kojic acid gene cluster in 2010. This study provides a better understanding of the roles of the MFS transporter and contributes to future studies on the kojic acid gene cluster in *A. oryzae*.

## 2. Materials and Methods

### 2.1. Strains and Media

*A. oryzae* 3.042 (CICC 40092) was used as the wild-type strain (WT) and the recipient strain for generating mutants. The yeast strain BY4741 was used for yeast transformation. *A. oryzae* was cultured on Czapek-Dox (CD) medium (2% soluble starch, 0.2% NaNO_3_, 0.1% KH_2_PO_4_, 0.05% MgSO_4_, 0.05% KCl, 0.05% NaCl, 0.002% FeSO_4_ and pH 5.5) to characterize the colonial morphologies. The modified CD medium (10% soluble starch, 0.2% NaNO_3_, 0.1% K_2_HPO_4_, 0.05% MgSO_4_, 0.05% KCl, 0.001% FeSO_4_, 0.1% yeast extract and pH 5.5) was utilized to produce kojic acid. A YPD (yeast extract/peptone/dextrose) medium (1% yeast extract, 2% peptone, 2% D-glucose) was used for the culture of yeast. The induction medium (0.67% yeast nitrogen base, 2% galactose, 0.078% DO Supplement–Ura (Code No. 630416, Clontech, Dalian, China) was used to express the AoKap4 protein in yeast.

### 2.2. Sequence Analysis of Aokap4

The conserved motifs of the AoKap4 protein were examined using SMART (http://smart.embl-heidelberg.de/ (accessed on 20 August 2022)) and MOTIF Search (https://www.genome.jp/tools/motif/ (accessed on 20 August 2022)). The transmembrane helices of the AoKap4 protein were predicted using TMHMM Server v.2.0 (https://services.healthtech.dtu.dk/service.php?TMHMM-2.0/ (accessed on 20 August 2022)). For phylogenetic tree analysis, the homologues of the AoKap4 protein were identified against the EnsemblFungi Database (http://fungi.ensembl.org/index.html (accessed on 20 August 2022)) with BLASTP. AoKap4 and its homologues were used to construct a phylogenetic tree using MEGA7 software. The parameters were as follows: neighbor-joining (NJ) method, 1000 replicates, Poisson model, uniform rates and pairwise deletion.

### 2.3. Mutagenesis of Aokap4 in A. oryzae

The *U6* promoter with the target sequence for *Aokap4* was amplified using the plasmid pPTRII-Cas9-AoGld3 [26] as a template with the primers PU6-F and PU6-Aokap4-R, generating the fragment PU6-Aokap4. The U6 terminator and sgRNA sequence were amplified from the vector pPTRII-Cas9-AoGld3 with the primers TU6-Aokap4-F and TU6-R, yielding the fragment Aokap4-TU6. The two fragments above were fused by the primers PU6-F and TU6-R, and inserted into the *SmaI* site of the pPTRII-Cas9 vector [27], yielding the plasmid pPTRII-Cas9-Aokap4. The constructed plasmid was transformed into the *A. oryzae* 3.042 strain. Transformants were screened on the CD medium containing pyrithiamine, and mutations in *Aokap4* were confirmed via nucleotide sequencing.

### 2.4. Phenotypic Characterization

To investigate the growth of fungal strains, fresh spores were collected from 5-day-old CD plates and adjusted to the concentration of 10^6^ spores/mL with 0.05% Triton X-100 solution. The spore suspensions were inoculated on CD agar medium for three days at 30 °C. The colonies were photographed and their mycelial diameters were measured. The spores were harvested from 6 mm cores using 2 mL of 0.05% Triton X-100 solution, and the numbers of spores were estimated using a hemocytometer. To determine the biomass of the fungal strains, the spore suspensions (10^6^ spores/mL) were spread on CD agar plates covered by cellophane. After cultivation for three days, the mycelia were collected and dried overnight at 60 °C to weight their biomass. Three replicates were analyzed for each assay.

### 2.5. Analysis of the Yields of Kojic Acid

The concentration of kojic acid was estimated using the colorimetric method [28]. Firstly, kojic acid with different concentrations (0–0.4 mg/mL) was mixed with the freshly prepared FeCl_3_-HCl solution (0.06 M FeCl_3_ and 0.27 M HCl) and the absorbance of the complexation was detected at 500 nm using a Multiskan GO Microplate Reader (Thermo Scientific, Waltham, MA, USA) to manufacture the standard curve. Then, a conidial suspension (10^6^ spores/mL) of the fungal strains was inoculated in the modified liquid CD media with shaking at 200 rpm and 30 °C for seven days. The supernatant was harvested to react with Fe^3+^ ions (FeCl_3_-HCl solution) to estimate the yield of kojic acid. The mycelia were collected and dried to quantify their biomass. The content of kojic acid was divided by the biomass. All experiments were repeated three times.

### 2.6. Gene Expression Analysis Using RT-qPCR

The total RNA was extracted from strains cultured on CD agar or liquid medium at 30 °C for four days using a Fungal Total RNA Extraction Kit (Omega, Norcross, GA, USA) according to the manufacturer’s protocol. The genomic DNA was removed using a gDNA Eraser (TaKaRa, Dalian, China). One μg of RNA was used to synthesize cDNA with a Prime Script™ RT reagent Kit (TaKaRa, Dalian, China). Quantitative real-time PCR was performed using a SYBR Green PCR Kit (TaKaRa, Dalian, China) and a CFX96 Real-Time PCR Detection System (Bio-Rad, Hercules, CA, USA) using the primers listed in Table S1. The histone H1 (AO090012000496) was used as a reference gene. The relative gene-expression levels were calculated using the method described previously [29]. All assays were carried out in triplicate.

### 2.7. Construction of Double-Deletion Mutant of Aokap4 and kojT

The constructed plasmid pPTRII-Cas9-Aokap4 was transformed into a *kojT*-deletion strain generated previously by us [27]. The transformants were subcultured on the CD medium containing pyrithiamine and verified via sequencing.

### 2.8. Overexpression of kojT in the Aokap4 Deletion Strain

The resulting plasmid pPTRII-Cas9-Aokap4 was introduced into the *kojT*-overexpression strain constructed previously by ourselves using the protoplast transformation method [30]. The candidates were selected using 0.1 μg/mL pyrithiamine and verified via DNA sequencing.

### 2.9. Heterologous Expression of AoKap4 in Yeast

The coding sequence of *Aokap4* was amplified with the primer pYES2-Aokap4-GFP-F/R, and inserted into the *BamHI* site of the pYES2-GFP vector [31]. The transformation plasmids pYES2-GFP and pYES2-Aokap4-GFP were introduced into the yeast strain BY4741 using the PEG/LiAc-based method. The transformants with the pYES2-GFP vector served as the control. After galactose induction at 28 °C for 12 h using 2% galactose as a carbon source, the yeast cells were collected to observe the fluorescence signal using ZOE™ Fluorescent Cell Imager (BIO-RAD, Hercules, CA, USA).

### 2.10. Statistical Analysis

All the data were processed using Excel 2020 (Microsoft Corp., Redmond, WA, USA). The statistical significance was determined using GraphPad Prism 8 (GraphPad Software, Inc., San Diego, CA, USA) based on Student’s *t*-tests. The values are the mean ± SD (*n* = 3).

## 3. Results

### 3.1. Identification and Characterization of Aokap4 Gene

**Figure 1:**
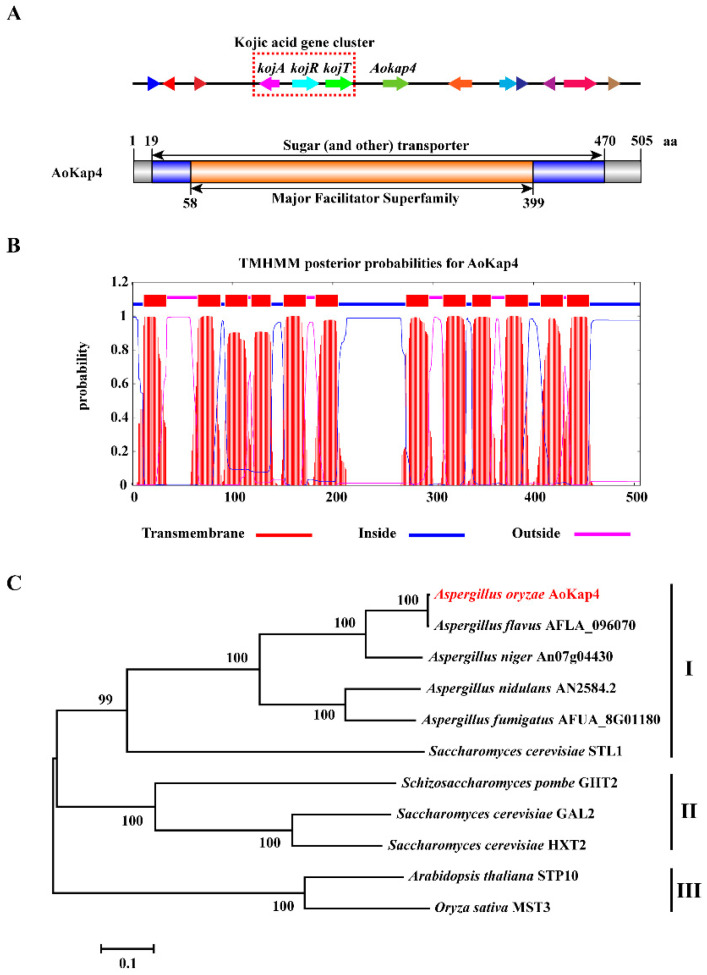
Sequence characterization and phylogenetic analysis of the AoKap4 protein. (**A**) The location of *Aokap4* in the kojic acid gene cluster and the domain analysis of the AoKap4 protein. The *Aokap4* gene adjacent to *kojT* within the kojic acid gene cluster encoded a major facilitator superfamily transporter. The kojic acid gene cluster is indicated in the red dashed box. (**B**) The analysis of transmembrane helices of the AoKap4 protein using the TMHMM Server. (**C**) Phylogenetic analysis of AoKap4 and its orthologs from fungi and plants. The phylogenetic tree was constructed using MEGA7 with 1000 bootstrap replicates and the neighbor-joining (NJ) method.

The *A. oryzae Aokap4* gene (AO090113000139) is located adjacent to *kojT* within the kojic acid gene cluster. The *Aokap4* gene contains an open reading frame (ORF) of 1578 bp, encoding a 505 amino acid residue. To explore the function of the AoKap4 protein, we firstly performed the functional domain analysis. The domain analysis results revealed that the AoKap4 protein harbored a domain of sugar transporter belonging to the major facilitator superfamily (MFS) (Figure 1A). The analysis of the transmembrane domain showed that AoKap4 contained twelve transmembrane domains across the entire MFS domain and the TC number of AoKap4 is 2.A.1.1, based on STL1 as the homologue of AoKap4 in *S. cerevisiae* (Figure 1B). To analyze the localization of AoKap4, the AoKap4-GFP fusion protein was expressed heterologously in the yeast strain BY4741. After galactose induction, the transformed yeast cells with the AoKap4-GFP fusion protein were observed at 12 h, and the green fluorescence signals of the AoKap4-GFP fusion protein were detected in the whole cell (Figure S1). To elucidate the phylogenetic relationship between AoKap4 and its orthologs, the phylogenetic tree was constructed. The phylogenetic analysis showed that AoKap4 and its orthologs were classified into three clades (I, II and III) (Figure 1C). AoKap4 and its orthologs of *Aspergillus* fell into clade I, while the transporter proteins derived from *Saccharomyces cerevisiae* clustered separately from those of *Aspergillus*, consisting of clade II (Figure 1C). However, the sugar transporter STL1 in *S. cerevisiae* was clustered with the members from *Aspergillus* (Figure 1C).

### 3.2. Aokap4 Affected the Growth of A. oryzae

**Figure 2:**
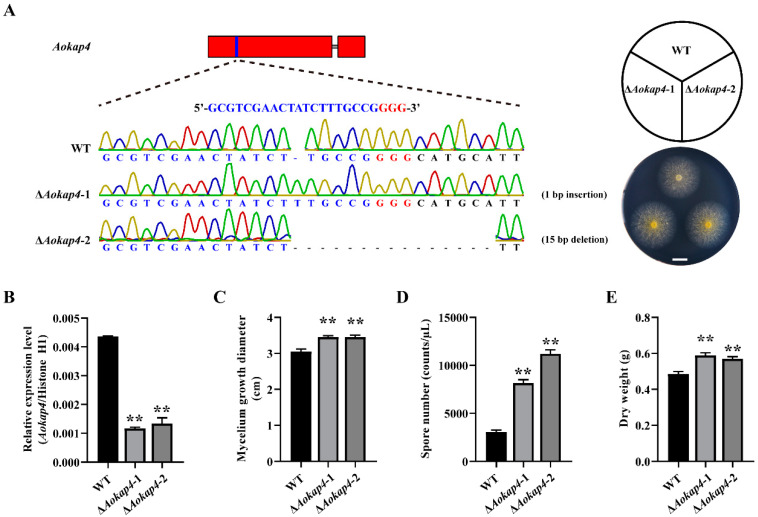
Effects of disruption of *Aokap4* on the growth of *A. oryzae*. (**A**) Construction of *Aokap4-*deletion mutants using the CRISPR/Cas9 system. The targeted sequence for *Aokap4* is shown in blue and the corresponding PAM sequence is indicated in red. The number and types of mutations are shown on the right. The *Aokap4-*deletion mutants Δ*Aokap4*-1 and Δ*Aokap4*-2 harbor a 1 bp insertion and a 15 bp deletion generated by CRISPR/Cas9, respectively. The wild-type (WT), Δ*Aokap4*-1 and Δ*Aokap4*-2 were grown on the Czapek-Dox (CD) agar medium for three days. (**B**) Transcript levels of *Aokap4* in the WT and *Aokap4-*deletion strains. The mycelia from these fungal strains, cultivated in the modified CD liquid medium for four days at 30 °C, were collected to isolate total RNA. The relative expression levels of the *Aokap4* gene were examined via qPCR based on three replicates. (**C**–**E**) Comparison of colony diameter (**C**), conidia formation (**D**) and biomass (**E**) in the WT and *Aokap4-*deletion strains. Asterisks indicate statistical significance in the differences between the WT and *Aokap4-*deletion strains based on Student’s *t*-tests (** *p* < 0.01). Scale bars = 1 cm.

To investigate AoKap4 function in *A. oryzae*, we mutated the *Aokap4* gene via the CRISPR/Cas9 system, and successfully obtained two *Aokap4*-disruption mutants (Δ*Aokap4*-1 and Δ*Aokap4*-2) (Figure 2A). In the Δ*Aokap4*-1 mutant, a 1 bp insertion occurred in the transcript of *Aokap4*, resulting in the termination of translation (Figure 2A). In the Δ*Aokap4*-2 strain, a 15 bp deletion was found in the coding sequence of *Aokap4*, which led to the loss of five amino acids in the sugar transporter domain of the AoKap4 protein (Figure 2A). These mutations caused a significant decline in the expression of *Aokap4* (Figure 2B). To study the effects of the disruption of *Aokap4* on the growth of *A. oryzae*, the wild-type and *Aokap4*-disrupted strains were inoculated on the CD agar plates, cultured for three days at 30 °C, and their growth characterization was analyzed and compared. The growth of the *Aokap4*-deletion mutants increased significantly compared to the wild-type strain (Figure 2C). The *Aokap4*-deletion mutants formed more conidia than the wild-type strain (Figure 2D). Meanwhile, the disruption of *Aokap4* resulted in a significant increase in the biomass relative to the control wild-type strain (Figure 2E).

### 3.3. Disruption of Aokap4 Impaired the Sensitivity to Stress

**Figure 3:**
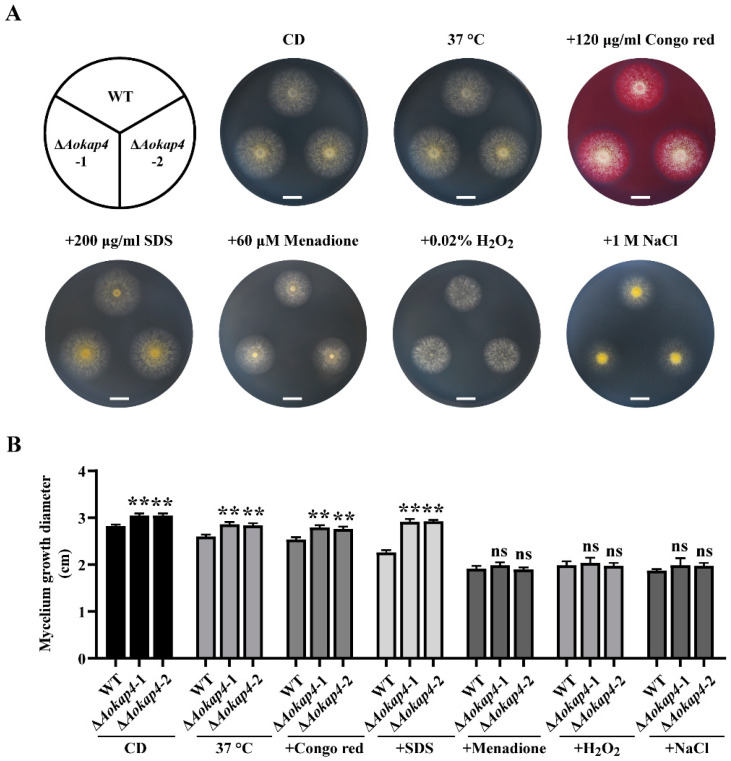
Phenotypical comparison of the wild-type and *Aokap4-*deletion strains under stress. (**A**) Colony growth of the wild-type and *Aokap4-*deletion strains on the CD agar medium with 120 μg/mL Congo red, 200 μg/mL SDS, 60 μg/mL menadione, 0.02% H_2_O_2_ and 1 M NaCl for three days at 30 °C. For heat stress, the plates were incubated at 37 °C. (**B**) Colony diameter of the wild-type and *Aokap4-*deletion strains under stress. The colony diameter of the wild-type and *Aokap4-*deletion strains cultivated on CD plates for three days under stress was measured. Asterisks indicate statistical significance in the differences between the WT and *Aokap4-*deletion strains based on Student’s *t*-tests (** *p* < 0.01; ns, no significant difference). Scale bars = 1 cm.

To further explore the role of *Aokap4* in *A. oryzae*, the heat- (37 °C), cell-wall- (Congo red and SDS), oxidative- (menadione and H_2_O_2_) and osmotic (NaCl)-stress tolerance of the *Aokap4* disruptants were analyzed. The wild-type and *Aokap4-*deletion strains were cultured on the agar medium with 120 μg/mL Congo red, 200 μg/mL SDS, 60 μg/mL menadione, 0.02% H_2_O_2_ or 1 M NaCl at 37 °C for three days to test their stress (Figure 3A). The *Aokap4* disruptants showed significant insensitivity against heat and cell-wall stress, and had a similar growth response to these stressors as the control treatment (Figure 3A,B), suggesting that deletion of *Aokap4* decreased the sensitivity to heat and cell-wall stress. However, under oxidative and osmotic stress, no significant differences in growth were observed between the wild-type strain and the Δ*Aokap4* mutants (Figure 3), indicating *Aokap4* is irrelevant to oxidative and osmotic stress.

### 3.4. Disruption of Aokap4 Repressed Kojic Acid Production in A. oryzae

**Figure 4:**
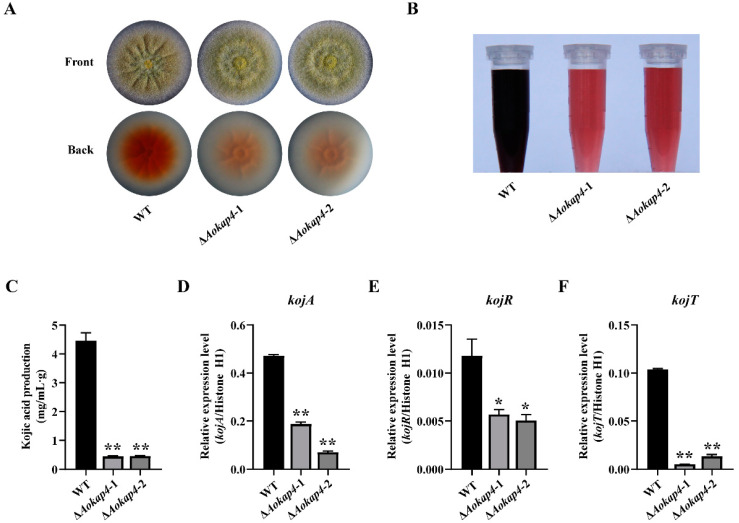
AoKap4 affected kojic acid production in *A. oryzae*. (**A**) Colony growth of the wild-type and *Aokap4-*deletion strains cultured on the CD plates with 1 mM FeCl_3_ for four days. Kojic acids chelated with FeCl_3_ showing a visible red. (**B**) Color reaction of kojic acid produced by the wild-type and *Aokap4-*deletion strains inoculated in the modified CD liquid medium for seven days at 200 rpm and 30 °C. (**C**) The yield of kojic acid in the wild-type and *Aokap4-*deletion strains cultivated in the modified CD liquid medium for seven days at 30 °C. (**D**–**F**) Transcript levels of *kojA* (**D**), *kojR* (**E**) and *kojT* (**F**) in the wild-type and *Aokap4-*deletion strains. The mycelia from the wild-type and *Aokap4-*deletion strains cultured in the modified CD liquid medium were harvested to extract total RNA. The gene-expression levels were detected via qPCR. All assays were performed in triplicate. Asterisks indicate statistical significance in the differences between the WT and *Aokap4-*deletion strains based on Student’s *t*-tests (** *p* < 0.01, * *p* < 0.05).

To investigate the influences of *Aokap4* on kojic acid production in *A. oryzae*, the yield of kojic acid produced by the wild-type and *Aokap4-*disruption strains was analyzed. Firstly, the wild-type and *Aokap4-*disruption strains were inoculated on the modified CD agar plates supplemented with 1 mM FeCl_3_ that were chelated with kojic acid, showing a visible red. After cultivation for three days at 30 °C, the *Aokap4*-disrupted mutants displayed a less-red color intensity than the control wild-type strain (Figure 4A), suggesting that the *Aokap4*-deletion strains produced less kojic acid than the wild-type strain. To quantify the amount of kojic acid, the wild-type and *Aokap4-*disruption strains were cultured in the modified CD liquid medium for seven days at 200 rpm and 30 °C. The amount of kojic acid in the Δ*Aokap4* mutants decreased by 92% compared to the wild-type strain (Figure 4B,C). To investigate whether the reduced kojic acid production of the *Aokap4*-deletion strains is associated with the genes of the kojic acid gene cluster, the expression levels of *kojA*, *kojR* and *kojT* were analyzed. In the Δ*Aokap4* mutants, the transcript levels of *kojA*, *kojR* and *kojT* were down-regulated significantly, consistent with the declined production of kojic acid (Figure 4D–F). These results indicate that *Aokap4* is required for kojic acid production of *A. oryzae*.

### 3.5. Knockout of kojT in the Aokap4-Disrupted Strain Impaired Kojic Acid Production

**Figure 5:**
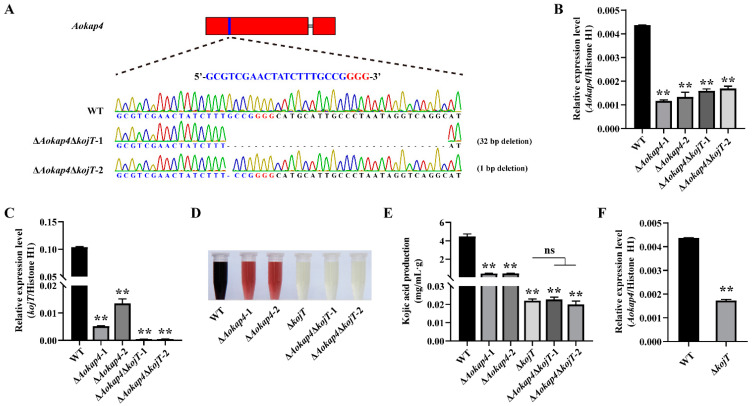
Disruption of *kojT* in *Aokap4-*deletion mutant inhibited kojic acid production. (**A**) Construction of the double-deletion mutants of *kojT* and *Aokap4* using a CRISPR/Cas9 system. The targeted position is highlighted by the blue box. The Cas9 targeted sequence for *Aokap4* is indicated in blue and the PAM sequence is in red. The number and type of mutants in the *Aokap4* gene are indicated on the right. (**B**,**C**) Transcriptional expression of *Aokap4* (**B**) and *kojT* (**C**) in the wild-type and double-deletion mutants of *kojT* and *Aokap4*. Total RNA was extracted from the mycelia cultivated in the modified CD liquid medium for four days at 30 °C. (**D**) Kojic acid produced by the wild-type (WT), *Aokap4-*deletion mutants (Δ*Aokap4*-1 and Δ*Aokap4*-2), *kojT*-disrupted strain (Δ*kojT*) and double-deletion mutants of *kojT* and *Aokap4* (Δ*Aokap4*Δ*kojT*-1 and Δ*Aokap4*Δ*kojT*-2) cultured in the modified CD liquid medium for seven days at 30 °C. (**E**) The amount of kojic acid generated by WT, Δ*Aokap4*-1, Δ*Aokap4*-2, Δ*kojT*, Δ*Aokap4*Δ*kojT*-1 and Δ*Aokap4*Δ*kojT*-2. These fungal strains were incubated in the modified CD liquid medium for seven days at 30 °C. (**F**) The mRNA level of *Aokap4* in the wild-type and *kojT-*deletion strains. ** *p* < 0.01 represents significant differences between the wild-type and mutants, and ns indicates no significant difference.

The data presented above indicate that the involvement of *Aokap4* in kojic acid biosynthesis is closely related to the kojic acid gene cluster. In the kojic acid gene cluster, *kojT* encodes an MSF transporter responsible for delivering kojic acid to the extracellular environment. *Aokap4* also belongs to the MSF transporter, which is adjacent to *kojT*. Given the reduced production of kojic acid in the *Aokap4*-deletion mutants, we reasoned that the knockout of *kojT* in the *Aokap4*-disrupted strain might severely impair kojic acid production. To this end, we constructed the double-deletion mutants of *Aokap4* and *kojT* (Δ*Aokap4*Δ*kojT*-1 and Δ*Aokap4*Δ*kojT*-2) based on the previously constructed *kojT*-deletion mutant using the CRISPR/Cas9 system (Figure 5A and Figure S2). Deletions of 32 bp and 1 bp occurred in the Δ*Aokap4*Δ*kojT*-1 and Δ*Aokap4*Δ*kojT*-2 double-deletion mutants, respectively (Figure 5A), which led to the premature translational termination of the AoKap4 protein. In the double-deletion mutants of *Aokap4* and *kojT*, mutations in *Aokap4* and *kojT* resulted in a significant decrease in the transcript levels of *Aokap4* and *kojT*, respectively (Figure 5B,C). The Quantification of kojic acid production revealed that the yield of kojic acid in the Δ*Aokap4*Δ*kojT*-1 and Δ*Aokap4*Δ*kojT*-2 double-deletion mutants had marked reductions and had declined to the same level as the Δ*kojT* mutant (Figure 5D,E). Additionally, the expression of *Aokap4* was down-regulated in the *kojT-*deletion mutant (Figure 5F).

### 3.6. Overexpression of kojT in Aokap4-Deletion Mutant Could Prevent the Reduced Production of Kojic Acid

**Figure 6:**
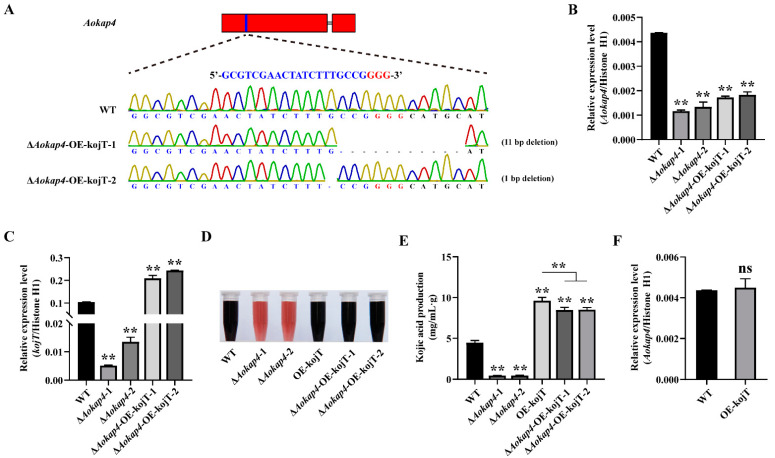
Overexpression of *kojT* in *Aokap4-*disrupted strain rescued the reduced production of kojic acid. (**A**) Targeted mutation of *Aokap4* in the *kojT*-overexpression strain using the CRISPR/Cas9 system, producing the Δ*Aokap4*-OE-kojT strain where *Aokap4* was deleted and *kojT* was overexpressed by *A. oryzae amyB* promoter. The targeted sequence of *Aokap4* is indicated in blue and the PAM is shown in red. The mutation types and the numbers of mutated sequences are shown on the right. (**B**,**C**) Transcript levels of *Aokap4* (**B**) and *kojT* (**C**) in the wild-type and Δ*Aokap4*-OE-kojT strains. (**D**) Color reaction of kojic acid with ferric ion, which is generated by WT, Δ*Aokap4*-1, Δ*Aokap4*-2, OE-kojT, Δ*Aokap4*-OE-kojT-1 and Δ*Aokap4*-OE-kojT-2. These strains were cultivated in the modified CD liquid medium for seven days. (**E**) The yield of kojic acid produced by WT, Δ*Aokap4*-1, Δ*Aokap4*-2, OE-kojT, Δ*Aokap4*-OE-kojT-1 and Δ*Aokap4*-OE-kojT-2 cultured in the modified CD liquid medium for seven days. (**F**) The expression level of *Aokap4* in the wild-type and *kojT-*overexpression strains. Asterisks indicate statistically significant differences between the wild-type and mutants (** *p* < 0.01). ns, no significant difference.

The above-mentioned data indicate that the deletion of *kojT* in the *Aokap4*-disrupted mutant inhibits kojic acid production more severely than the single mutation in the *Aokap4* gene. We therefore reasoned that the overexpression of *kojT* in the *Aokap4-*disrupted mutant might reverse the reduced production of kojic acid. To this end, we generated the Δ*Aokap4*-OE-kojT strains (Δ*Aokap4*-OE-kojT-1 and Δ*Aokap4*-OE-kojT-2) via the knockout of *Aokap4* in the *kojT*-overexpression strain using the CRISPR/Cas9 system (Figure 6A and Figure S3), in which *Aokap4* was deleted and *kojT* was overexpressed by the *A. oryzae amyB* promoter (Figure 6C). An 11 bp deletion in the *Aokap4* gene was confirmed in the Δ*Aokap4*-OE-kojT-1 strain (Figure 6A). The Δ*Aokap4*-OE-kojT-2 strain harbored a 1 bp deletion in the target sequence of the *Aokap4* gene (Figure 6A). Both mutations caused the decreased expression of *Aokap4* (Figure 6B). To detect the yield of kojic acid in the Δ*Aokap4*-OE-kojT strains, the wild-type, *Aokap4-*deletion mutants, *kojT*-overexpression strain and Δ*Aokap4*-OE-kojT strains were cultured in the modified CD liquid medium for seven days at 30 °C. The results showed the Δ*Aokap4*-OE-kojT strains recovered kojic acid production but did not return to the level of the *kojT*-overexpression strain (Figure 6D,E). Meanwhile, the mRNA level of *Aokap4* had no change in the *kojT*-overexpression strain compared with the control wild-type strain (Figure 6F).

## 4. Discussion

It was identified, in 2010, that the kojic acid gene cluster contains three genes: *kojA*, *kojR* and *kojT* [7]. However, to date, there is no information on the function of the genes near the kojic acid gene cluster or whether the genes near the kojic acid gene cluster affect kojic acid production. Here, a novel MSF transporter gene, *Aokap4*, near the kojic acid gene cluster, was found to involve kojic acid production, which worked in cooperation with KojT. The deletion of *Aokap4* contributed to mycelium growth and conidia formation, and resulted in enhanced heat- and cell-wall-stress tolerance in *A. oryzae*, suggesting that AoKap4 is required for growth and stress tolerance in *A. oryzae*.

The development of aspergilli primarily includes the growth of the vegetative mycelium and spore formation [32,33,34]. In this present study, the deletion of *Aokap4* contributed to mycelium growth and conidia formation, together with increased biomass (Figure 2), suggesting that AoKap4 acts as a repressor of the growth of *A. oryzae*. Considering the reduced kojic acid production in the *Aokap4*-disrupted strain, it seems that AoKap4 is involved in antagonistic action between growth and kojic acid production. The antagonistic action appears to be widespread [9,13,35]. For example, the deletion of *kpeA* in *A. oryzae* inhibits conidia formation but increases kojic acid production, while overexpression of *Aokap2* suppresses mycelium growth and conidia formation but contributes to kojic acid production [9,13]; the knockout of *msnA* in *A. parasiticus* and *A. flavus* represses colony growth but enhances kojic acid synthesis [35]. As an ortholog of *Aokap4*, the *STL1* gene encodes a sugar transporter-like protein of *S. cerevisiae*, belonging to the MSF transporter [25]. Although the phylogenetic analysis revealed that AoKap4 is clustered with STL1 of *S. cerevisiae* (Figure 1C), the role of AoKap4 in the growth of *A. oryzae* may be different from that of STL1, which is supported by the finding that the disruption of *STL1* does not influence on the growth of *S. cerevisiae* [25]. Later research found that *STL1* is involved in stress adaptation [24,36,37,38]. Likewise, the disruption of *Aokap4* decreased the sensitivity to heat and cell-wall stress, but not oxidative and osmotic stress (Figure 3), indicating that *Aokap4* is required for tolerance to heat and cell-wall stress and unrelated to oxidative and osmotic stress. Furthermore, in general, post-translational regulation is involved in the response to environmental stresses, such as phosphorylation, which is the most common method of post-translational regulation [39,40]. In *A. nidulans*, the HogA (high-osmolarity glycerol) kinase and histidine kinases participate in the response to stress by phosphorylating transcription factors [39,40,41]. This means that AoKap4 post-translational regulation may be closely related to HogA-dependent and His-kinase pathways. Collectively, these results suggest that AoKap4 plays multiple roles in growth, and in the heat- and cell-wall-stress responses, as well as kojic acid synthesis.

The kojic acid gene cluster is a key point in producing kojic acid, and includes *kojA*, *kojR* and *kojT*. The transcription factor KojR activates the FAD-dependent oxidoreductase KojA and the MFS transporter KojT to regulate kojic acid biosynthesis [8]. The deletion of *kojT* did not lead to the complete loss of kojic acid production, implying that there are other transporters that function in kojic acid production [7]. Here, a novel MSF transporter, AoKap4, was found to involve kojic acid production in *A. oryzae*. The disruption of *Aokap4* inhibited, but did not abolish, kojic acid production with the decreased expression of *kojA*, *kojR* and *kojT*; this indicates that there is a redundant gene with functions the same as or similar to those of *Aokap4* in kojic acid production, and the involvement of *Aokap4* in kojic acid production is closely related to the kojic acid gene cluster. Almost all of the kojic acid-related genes are associated with the kojic acid gene cluster. The increased expression of *kojA* and *kojR* is responsible for enhanced kojic acid production, caused by the deletion of the transcriptional regular protein gene *kpeA* [9]. The expression of *kojA*, *kojR* and *kojT* decreases in the *laeA* disruption strain, which is deprived of kojic acid production [4]. The disruption of the nitrate transporter gene *nrtA* releases the inhibition of nitrate to kojic acid production and contributes to kojic acid production in the presence of nitrate coupled with the increased expression of *kojA*, *kojR* and *kojT* [5]. Knocking down the expression of the ZIP transporter gene *AoZip2* inhibits the production of kojic acid with the reduced expression of *kojA*, *kojR* and *kojT* [12]. However, the correlation between the identified kojic acid-related genes and the kojic acid gene cluster is verified merely via transcriptional analysis, but not genetic analysis. In this study, the deletion of *Aokap4* resulted in the decreased expression of *kojT,* while the disruption of *kojT* caused the declined expression of *Aokap4* (Figure 4F and Figure 5F), implying that there might be an interaction between *Aokap4* and *kojT* to function in kojic acid production in *A. oryzae*. However, *Aokap4* might not keep pace with *kojT* in enhancing kojic acid production, which is supported by the finding that the expression of *Aokap4* has no change in the *kojT*-overexpression strain (Figure 6F). Furthermore, the amount of kojic acid production in the *Aokap4*-deletion mutants decreased by 92% compared with the wild-type strain, while the production of kojic acid in the double-deletion mutant of *Aokap4* and *kojT* was less than 5% of that in the *Aokap4*-deletion mutants, suggesting that *Aokap4* and *kojT* are essential for kojic acid production in *A. oryzae*. Moreover, the knockout of *kojT* in the *Aokap4*-disrupted strain significantly inhibited kojic acid production of the *Aokap4*-disrupted strain and led to a decline in kojic acid production in the double-deletion mutants of *Aokap4* and *kojT* to the same level as that of the *kojT*-deletion mutant (Figure 5D,E); meanwhile, the overexpression of *kojT* in the *Aokap4* disruptant could recover the declined production of kojic acid (Figure 6D,E), indicating that *kojT* genetically acts downstream of *Aokap4*.

Interestingly, kojic acid production in the Δ*Aokap4*-OE-kojT strain was reversed by overexpressing *kojT*, but did not reach the same level as the OE-kojT strain (Figure 6D,E), indicating that the overexpression of *kojT* can complement the phenotype of the Δ*Aokap4* mutant with reduced kojic acid production, but *Aokap4* did not have the same role in kojic acid production as *kojT*. In *A. oryzae*, the MSF transporter KojT is predicted to be located on the cell membrane and the transport kojic acid to the extracellular environment [7]. Given that *Aokap4* acts upstream of *kojT*, the novel MSF transporter AoKap4 may not be located on the cell membrane and may function in delivering an intermediate of kojic acid biosynthesis, but not kojic acid; this is supported by the result wherein the AoKap4-GFP fusion protein was expressed in the yeast. After galactose induction for 12 h, the green fluorescence signal was detected in the entire yeast cell, suggesting that AoKap4 is localized in the cytoplasm, and the localization of AoKap4 to the plasma membrane cannot be excluded (Figure S1). Consistent with this finding is the fact that STL1, the ortholog of AoKap4 in *S. cerevisiae*, is a dual-targeted protein localized to the plasma membrane and vacuolar lumen [24]. Further analyses for the subcellular localization of AoKap4 will be necessary to analyze the role of *A. oryzae* in kojic acid production in detail.

In conclusion, a novel MSF transporter gene, *Aokap4*, located near the kojic acid gene cluster was identified and characterized as being involved in growth and kojic acid production. *Aokap4* negatively modulates mycelium growth and conidial formation and is required for heat and cell-wall stress tolerance in *A. oryzae*. *Aokap4* is involved in kojic acid production in *A. oryzae*, genetically acting upstream of *kojT*. The study provides a better understanding of the role of the MSF transporter in kojic acid production in *A. oryzae*, and lays a foundation for further research into the kojic acid gene cluster of *A. oryzae*.

## Data Availability

Not applicable.

## Supplementary Materials

The following supporting information can be downloaded at: https://www.mdpi.com/article/10.3390/jof8080885/s1, Figure S1: Heterologous expression of AoKap4 in yeast; Figure S2: Construction of the *kojT*-deletion mutant. Mutations in kojT; Figure S3: Generation of the *kojT*-overexpression strain; Table S1: Primers used in this study.
