# Supplementary material for: A Novel Major Facilitator Superfamily Transporter Gene Aokap4 near the Kojic Acid Gene Cluster Is Involved in Growth and Kojic Acid Production in Aspergillus oryzae
Source: J Fungi (Basel). 2022 Aug 22;8(8):885. doi: 10.3390/jof8080885 (PMC9410421; doi:10.3390/jof8080885)
Supplement: Supplementary file 1 [file jof-08-00885-s001.zip › jof-1853425-supplementary.pdf]

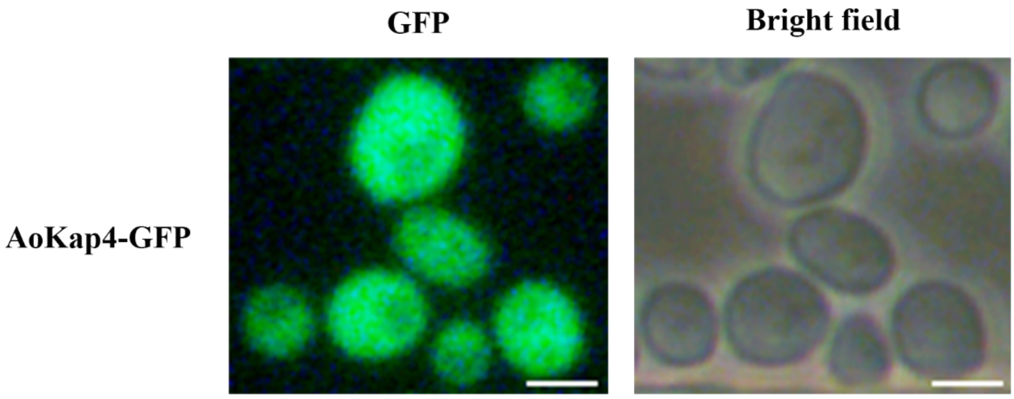

Figure S1. Heterologous expression of *AoKap4* in yeast. The green fluorescence signals of GFP and AoKap4-GFP infusion protein were detected after galactose induction at 28 °C for 12 h. Scale bars = 4 μm

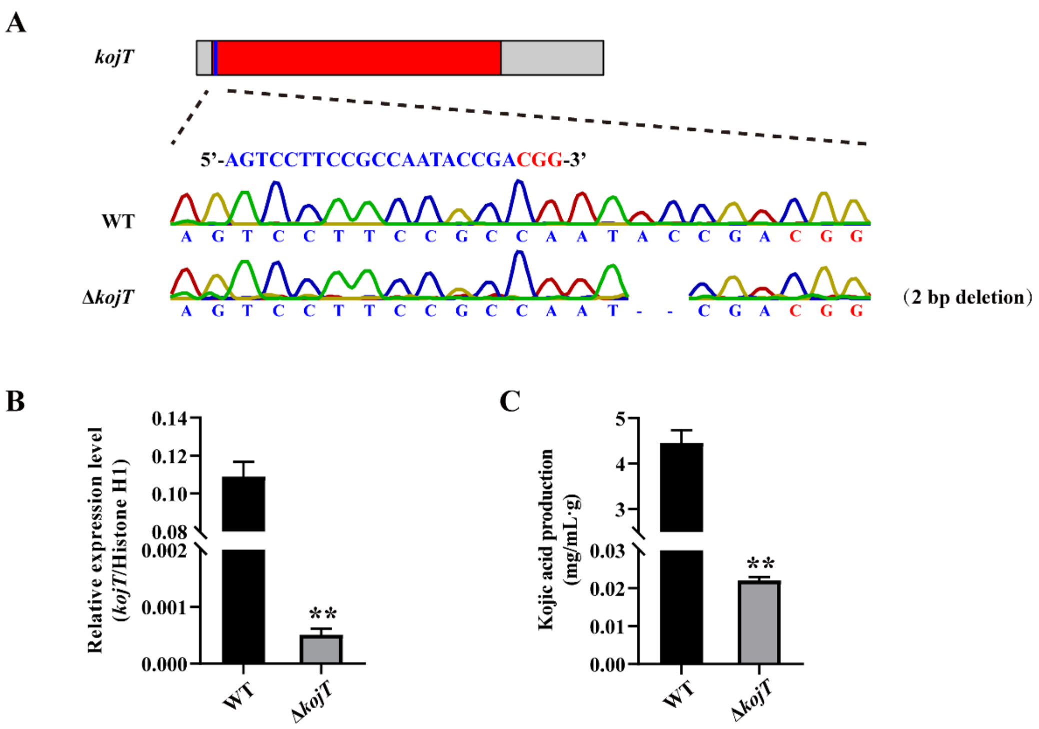

Figure S2. Construction of the *kojT*-deletion mutant. Mutations in *kojT* (**A**). The sgRNA sequence for *kojT* is indicated in blue. The protospacer adjacent motif (PAM) is underlined. The mutation type and the number of mutation sequences are shown on the right. The expression of *kojT* (**B**) was detected via qPCR in the *kojT*-deletion mutant. Total RNAs were isolated from the mycelia cultured in the modified CD medium for four days with shaking at 200 rpm and at 30 °C. (**C**) The yields of kojic acid in wild-type (WT) and *kojT*-deletion strains. Three independent experiments were performed. Asterisks indicate significant differences between the wild-type and *kojT*-deletion strains based on Student's t-tests (**$p < 0.01$).

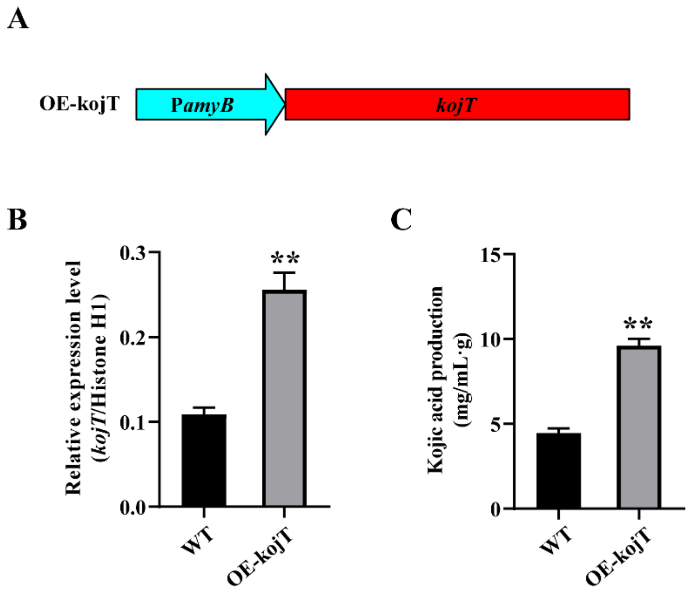

Figure S3. Generation of the *kojT*-overexpression strain. *KojT* were overexpressed by *A. oryzae amyB* promoter (P*amyB*). The expression of *kojT* was determined via qPCR. The mycelia cultivated for four days in the modified CD liquid medium were harvested to extract RNA. Three independent experiments were carried out. Asterisks indicate significant differences between the wild-type and OE-kojT strains based on Student's t-tests (**$p < 0.01$)

Table S1. Primers used in this study

Primer name	Primer sequence (5'-3')	Description
PU6-Aokap4-R	CGGCAAAGATAGTTCGACGCACTTGTTCTTCTTTACAATGATTTATTTA	*Aokap4* deletion
TU6-Aokap4-F	GCGTCGAACTATCTTTGCCGGTTTTAGAGCTAGAAATAGCAAGTTAAA	
PU6-F	CGACTCTAGAGGATCCCCGGGTAATGCCGGCTCATTCAAA	
TU6-R	AATTCGAGCTCGGTACCCGGGAGCAGCTCTATATCACGTGACG	
PU6-kojT-R	GAGCACATGCCTCGAGTATGAAGAGGCAGGTAGTTATAGTCTAG	*kojT* deletion
TU6-kojiT-F	AGTCCTTCCGCCAATACCGAGTTTTAGAGCTAGAAATAGCAAGTTAAA	
pEX2B-kojT-F	CGTGCCCGTGCTTAAGATGCAGTCCTTCCGCCAATA	*kojT*
pEX2B-kojT-R	AACGTTAAGTGGATCCTCAGGGGGTCTTTTTATTCCG	overexpression
pYES2-Aokap4-GFP-F	TACCGAGCTCGGATCCATGCAAGGCGAGGCACTT	Heterologous
pYES2-Aokap4-GFP-R	TGCTCACCATGGATCCTTCAATCTCTTCAACTATTTTCTTTCC	expression
rAokap4-F	TCGCGAATCAGTGGCTATGGATG	qPCR
rAokap4-R	GCCAACGACGGTGAATACGATG	
rkojA-F	ACACAAACGAGCCCCTTCAG	qPCR
rkojA-R	CCTCGTGACGGTCGAATGA	
rkojR-F	CAACTCAGGCACCGCTTTC	qPCR
rkojR-R	TCCAGCTAAACCCGTACACCT	
rkojT-F	TTTGGAGGATGCCGACGAT	qPCR
rkojT-R	TTCCTGGCGTGGAGTATGG	
rHistone-F	GACAACATCCAGGGTATCACTAAGC	qPCR
rHistone-R	GGTCTCCTCGTAGATCATGGCA	
CRISPR-S-Aokap4-F	ATGCAAGGCGAGGCACTT	Target sequence
CRISPR-S-Aokap4-R	TCATTCAATCTCTTCAACTATTTTCTT	detection
